# The risk for bloodstream infections is associated with increased parenteral caloric intake in patients receiving parenteral nutrition

**DOI:** 10.1186/cc6167

**Published:** 2007-10-24

**Authors:** Sharmila Dissanaike, Marilyn Shelton, Keir Warner, Grant E O'Keefe

**Affiliations:** 1 Harborview Medical Center, 325 9^th ^Ave, Seattle, WA 98104, USA; 2 Department of Surgery, Texas Tech University Health Sciences Center, 3601 4^th ^St Lubbock, TX 79430, USA

## Abstract

**Background:**

Patients receiving total parenteral nutrition (TPN) are at high risk for bloodstream infections (BSI). The notion that intravenous calories and glucose lead to hyperglycemia, which in turn contributes to BSI risk, is widely held but is unproven. We therefore sought to determine the role that hyperglycemia and parenteral calories play in the development of BSI in hospitalized patients receiving TPN.

**Methods:**

Two hundred consecutive patients initiated on TPN between June 2004 and August 2005 were prospectively studied. Information was collected on patient age, sex, admission diagnosis, baseline laboratory values, intensive care unit (ICU) status and indication for TPN. Patients in the ICU were managed with strict glycemic control, whereas control on the general ward was more liberal. The maximum blood glucose level over each 8-hour period was recorded, as were parenteral daily intake, enteral daily intake and total daily caloric intake. The primary outcome measure was the incidence of BSI. Additional endpoints were ICU length of stay, hospital length of stay and mortality.

**Results:**

A total of 78 patients (39%) developed at least one BSI, which were more common in ICU patients than in other hospitalized patients (60/122 patients versus 18/78 patients; *P *< 0.001). Maximum daily blood glucose concentrations were similar in patients with BSI and in patients without BSI (197 mg/dl versus 196 mg/dl, respectively). Patients with BSI received more calories parenterally than patients without BSI (36 kcal/kg/day versus 31 kcal/kg/day, *P *= 0.003). Increased maximum parenteral calories, increased average parenteral calories, and treatment in the ICU were strong risk factors for developing BSI. There was no difference in mortality between patients with and without BSI.

**Conclusion:**

Increased parenteral caloric intake is an independent risk factor for BSI in patients receiving TPN. This association appears unrelated to hyperglycemia. Based upon our observations, we suggest that parenteral caloric intake be prescribed and adjusted judiciously with care taken to account for all intravenous caloric sources and to avoid even short periods of increased intake.

## Introduction

Total parenteral nutrition (TPN) can be a valuable adjunct in providing nutrition to hospitalized patients. Reviews of surgical patients receiving perioperative TPN have shown a reduction in morbidity in severely malnourished patients [1,2]. A meta-analysis of nine randomized trials showed an aggregate mortality benefit in critically ill patients on TPN [3], despite a 1.7-fold increase in infectious complications. Other studies have shown an increased infection risk without a survival benefit in patients receiving TPN [4-7]. There is a reported sepsis incidence of between 20% and 30% in patients receiving parenteral nutrition [8-11]. The high risk of sepsis is a major factor leading to an overall preference for enteral nutrition over parenteral nutrition.

Tight glycemic control has been demonstrated to reduce mortality in critically ill surgical patients and to limit certain morbidities (acute renal failure, for example) in critically ill medical patients [12,13]. Patients experiencing the stress of trauma, critical illness or major surgery typically display endogenous insulin resistance that is characterized by reduced insulin uptake in peripheral tissues, along with an increase in glucose production. When combined with a large exogenous dextrose load such as occurs with TPN, the glucose oxidation capacity can easily be exceeded, which predisposes patients to develop significant hyperglycemia [14-16]. It is thought that hyperglycemia contributes to adverse outcomes associated with TPN in critically ill patients and other hospitalized patients.

Hyperglycemia is associated with an increased incidence of bloodstream infections (BSI) and sepsis in surgical patients. In one study, adverse outcomes in a cohort of 111 critically ill patients were attributed to TPN-associated hyperglycemia [10,17]. This risk is not restricted to surgical patients. Patients receiving stem cell transplantation have an increased infection risk with TPN-associated hyperglycemia [18]. In contrast to these reports, others have suggested that the amount of intravenous glucose rather than hyperglycemia is detrimental [19].

Herein, we sought to determine whether hyperglycemia, in the context of contemporary approaches to glycemic control, was associated with BSI in hospitalized patients receiving TPN. We also investigated whether the amount of enteral and parenteral calories were associated with BSI risk. We hypothesized that patients developing BSI while receiving TPN had higher blood glucose (BG) concentrations and received more calories intravenously than patients who did not develop BSI.

## Materials and methods

### Study design, patient enrollment and data collection

The University of Washington institutional review board for human research approved the study protocol and waived the need for informed consent. Two hundred consecutive patients admitted to Harborview Medical Center between July 2004 and August 2005 who received TPN at any time during their hospitalization were prospectively followed. Details regarding patient age, sex, admission diagnosis, comorbid conditions, reason for TPN, hospital location (intensive care unit (ICU) versus general ward) and length of stay were collected. Nutritional parameters, including prealbumin, albumin and C-reactive protein, were measured at the discretion of the attending physicians and dietitians involved in the patients' care.

A catheter-related infection was defined as a positive culture from the catheter tip and a simultaneous blood culture positive for the same organism. Bacteremia was defined as a positive blood culture in a patient with clinical signs of infection.

We recorded the daily parenteral caloric intake and the daily enteral caloric intake for the duration of TPN administration. The maximum parenteral calories received during a 24-hour period were recorded for each patient. This represents the highest calorie load at any time during the study. The average daily parenteral caloric intake for each patient was calculated as the mean number of parenteral calories per day that the patient received. Sources such as intravenous medications diluted in dextrose, and intravenous lipids such as propofol infused for sedation, were included in the total parenteral calorie counts. Where patients received concurrent enteral nutrition, details regarding the maximum and average daily enteral calories were recorded. Total calories are the daily sum of enteral calories and parenteral calories.

The maximum BG over each 8-hour period was collected, giving each patient three daily BG measurements. The average BG and the maximum BG while receiving TPN were calculated. In patients who developed BSI, only glucose values and daily caloric totals prior to the infection were used. Patients were followed from the time TPN was initiated at least until discharge from the hospital. We included the time after discharge from hospital in our follow-up period where this information was available. We therefore documented inhospital mortality and overall mortality over the follow-up period.

### Details of patient care

The decision to initiate TPN was made by the attending surgeon or physician, independent of the present study. All patients received parenteral nutrition via a central venous access. Patients receiving TPN were assessed on a daily basis by a specialist clinical pharmacist and dietitian. Where enteral nutrition was used concurrently, the decision to start enteral feeding as well as the rate and volume of advancement was at the discretion of the attending physician. The type of formula and the goal rate was chosen in consultation with a dietitian.

Caloric requirements were initially calculated based on the Harris–Benedict equation with stress adjustments [20]. In several cases these estimates were refined following metabolic cart and nitrogen balance measurements. The dry weight, estimated by subtracting crystalloid resuscitation volumes from the measured weight, was generally used to determine caloric needs in patients with body mass index (BMI) < 30 kg/m^2^. An adjusted weight was used to calculate caloric needs in patients with BMI > 30 kg/m^2^. This was calculated as the average of the predicted body weight and the measured body weight. The predicted weight was calculated as follows: 50 ± 0.91 kg (height = 152.4 cm) for men and 45.5 ± 0.91 kg (height = 152.4 cm) for women [21].

A concentrated TPN formulation was used to minimize fluid loading. This consisted of approximately 50% of calories from carbohydrate infused at a rate of 3–5 mg/kg/min, 20% of calories from protein (1.5–2 g/kg) and 30% or less of calories from intravenous lipids. The two-in-one carbohydrate and protein solution was infused over 24 hours. Carbohydrates were provided as dextrose, and two commercial protein formulas (Travisol 10% and Clinisol 15%; Baxter Healthcare Corp. Deerfield, Illinois, USA) provided essential and nonessential amino acids. The lipid formulas (Intralipid 10% and Intralipid 20%; Baxter Healthcare Corp) were individual 250 ml or 500 ml containers of soybean-oil based emulsion, and they consisted primarily of long-chain triglycerides. Lipids were infused separately and limited to 12 hours each night, in order to reduce the known risk of proliferation of pathogenic organisms within the lipid emulsion [22,23].

TPN was infused at a steady rate with no adjustments or gradual rate increases during the initial period. The total calories prescribed were not changed based on adjustments in the estimation of nitrogen requirements. There was no defined protocol to wean TPN once the patient was able to tolerate an enteral diet, and TPN was discontinued at physician discretion.

Routine catheter exchanges were not used in the ICU or on acute care wards. Catheters were exchanged over a wire or removed completely at physician discretion, usually on clinical suspicion of infection. All catheter tips were cultured after removal.

### Protocol for glycemic control

Patients were treated with an intravenous insulin infusion while in the ICU, with a BG goal of 80–110 mg/dl. Hourly measurements and rate adjustments were performed until this range was achieved. All BG measurements were made using the AccuChek Inform bedside glucose measurement system (Roche Diagnostics, Basel, Switzerland). Once patients had achieved a stable BG level, the frequency of measurements was gradually decreased. Patients in the general wards had BG checks every 6 hours, with a BG goal below 150 mg/dl. Subcutaneous insulin dosed according to a sliding scale was used to treat elevated BG.

### Statistical analysis

The primary endpoint was the development of any BSI, which included either bacteremia or a catheter-related infection. Categorical data are presented as proportions (with percentages), and continuous variables are presented as medians with the associated interquartile range (25th–75th percentile). We compared the maximum BG concentration (single highest recorded value) and the maximum daily enteral caloric intake, parenteral caloric intake and total caloric intake between patients with and without BSI using the Mann–Whitney U test. Analysis of variance was used to adjust for multiple factors when comparing BG concentrations and caloric intake in patients with and without BSI. Chi-squared analysis was used to test associations between categorical data. All *P *values were two-tailed and actual values are presented. Finally, we used logistic regression to analyze the effects of multiple potential risk factors on the development of BSI. Adjusted odds ratios are presented with 95% confidence intervals from this analysis. Statistical analyses were performed with SPSS version 11.0 software (SPSS Inc., Chicago, IL, USA) and STATA version 8.2 software (STATACorp LP, College Station, TX, USA).

## Results

### Description of study cohort

**Figure 1:**
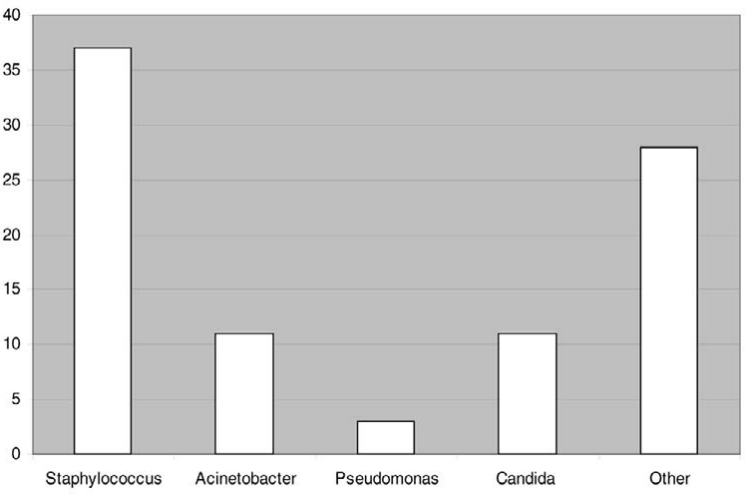
Organisms responsible for bloodstream infections. Staphylococci were responsible for approximately 50% of the bloodstream infections. Acinetobacter was the most common Gram-negative organism isolated.

Demographic data for the entire cohort are presented in Table 1. A total of 78 patients developed one or more BSI. There were no differences in age, sex or admission diagnosis between patients with and without BSI. Malignancy, documented immune suppression or infection as the admission diagnosis was not associated with an increased risk for BSI. Diagnosis of BSI was a median of 7.5 days (3–14 days) after initiation of TPN. As expected, ICU admission was an important risk factor for BSI (odds ratio, 2.9; 95% confidence interval, 1.5–5.6) (*P *< 0.001). Figure 1 shows the predominant isolated organisms. Staphylococcal species were responsible for 48% of infections. Fungal infections (*Candida albicans *or *Candida glabrata*) occurred in 16% of cases.

**Table 1:** Patient characteristics, diagnoses and baseline laboratory values

Characteristic	BSI-positive (*n *= 78)	BSI-negative (*n *= 122)	*P *value
Age (years)	55 (44–64)	55 (45–68)	0.2
Male gender	53 (68)	69 (57)	0.13
Intensive care unit location	60 (77)	62 (51)	0.0002
Admission diagnosis			
Trauma	26 (33)	29 (24)	0.15
Infection	17 (22)	18 (15)	0.45
Other general surgery diagnosis	25 (32)	51 (42)	0.18
Comorbid conditions			
Diabetes mellitus	12 (15)	17 (14)	0.84
Malignancy	10 (13)	17 (14)	1
Immunosuppression	9 (12)	20 (16)	0.41
Baseline nutrition laboratory values			
Prealbumin (g/dl)	7.2 (4.5–10.5)	8.6 (5.2–11.8)	0.25
Albumin (g/dl)	1.6 (1.3–2)	1.9 (1.5–2.5)	0.0009
C-reactive protein	140 (82–223)	144 (74–201)	0.5

Data presented as the median (interquartile range) or as *n *(%). BSI, bloodstream infections.

The indications for commencing TPN, the time spent with no oral intake prior to TPN, the duration of TPN and the duration of enteral feeding are presented in Table 2. The presence of an open abdomen and a lack of suitable enteral access were the most common indications for starting TPN. The duration of TPN and enteral nutrition were greater in patients with BSI.

**Table 2:** Details of parenteral nutrition and enteral nutrition administration

	BSI-positive (*n *= 78)	BSI-negative (*n *= 122)	*P *value
Indication for total parenteral nutrition			
Open abdomen	12 (15)	17 (14)	0.84
Lack of enteral access (post-pyloric)	8 (10)	20 (16)	0.29
Intolerance of enteral feeds at goal rate	19 (7)	8 (1)	0.0005
Ileus	7 (9)	16 (13)	0.49
Other	32 (41)	61 (50)	
Time until any form of nutrition started (days)	5 (3–7)	5 (3–7)	0.21
Duration of parenteral nutrition (days)	9 (6–21)	7 (4–11)	0.0001
Duration of enteral feeding (days)	8 (6–20)	6 (3–10)	0.0001

Data presented as the median (interquartile range) or as *n *(%). BSI, bloodstream infections.

Table 3 describes the hospital length of stay, the ICU length of stay, the follow-up period and mortality for our cohort. The median follow-up was 100 days. Patients who developed BSI had a longer ICU length of stay and hospital length of stay but did not have a higher fatality rate.

**Table 3:** Intensive care unit length of stay, hospital length of stay and mortality

	BSI-positive (*n *= 78)	BSI-negative (*n *= 122)	*P *value
Intensive care unit length of stay (days)	15 (15–31)	3.5 (0–11)	0.0001
Hospital length of stay (days)	33 (22–47)	19 (13–28)	0.0001
Follow-up (days)	117 (34–320)	76 (25–264)	0.12
Mortality	23 (30)	39 (32)	0.76

Data presented as the median (interquartile range) or as *n *(%). BSI, bloodstream infections.

### Analysis of factors potentially associated with bloodstream infections

**Figure 2:**
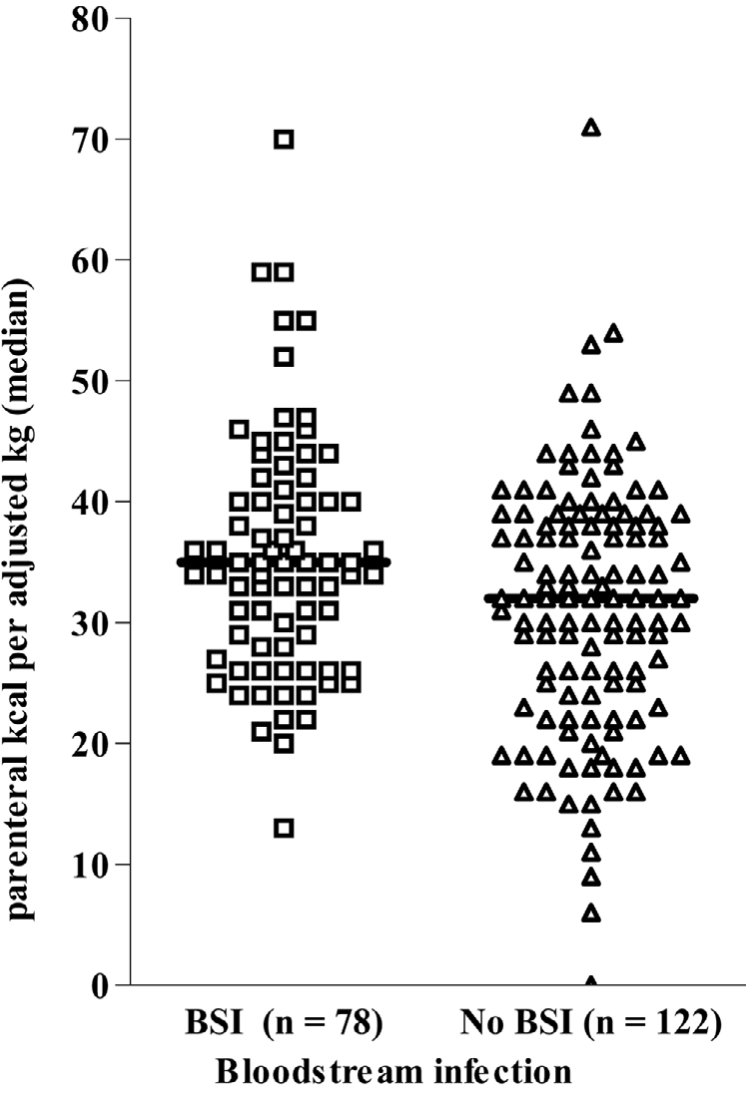
Maximum daily parenteral calories. Maximum parenteral calories over 24 hours for patients with and without subsequent bloodstream infections (BSI). Horizontal line, median for each group. As seen, patients who developed BSI did receive approximately 5 kcal/kg more per day than patients without BSI.

Patients received a wide range of parenteral calories, 70 kcal/kg/day being the reported maximum parenteral intake. The maximum daily parenteral caloric intake was higher in patients with BSI than in patients without BSI (36 kcal/kg versus 31 kcal/kg, respectively). These data are shown in Figure 2.

**Figure 3:**
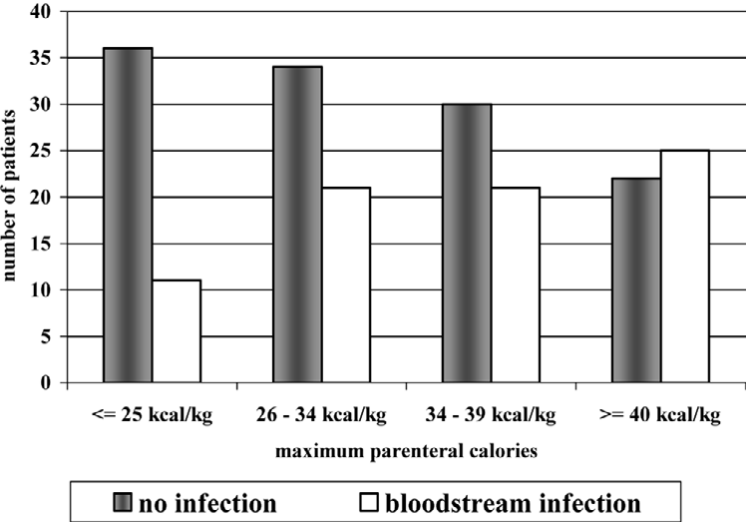
Occurrence of bloodstream infections according to quartile of maximum daily parenteral caloric intake. Number of patients with and without bloodstream infections plotted on the *y *axis against the quartile of maximum parenteral caloric intake on the *x *axis. There is a progressive increase in the proportion of patients with bloodstream infections with increased caloric intake.

We considered additional factors potentially related to BSI (hospital location, sex, BMI and whether the patient received any enteral support) using logistic regression. This analysis demonstrated a similar association between parenteral caloric intake and BSI that was similar to the unadjusted analysis. There was a 1.6-fold (95% confidence interval, 1.2–2.0) increase in BSI with each quartile increase in maximum parenteral calories after adjusting for ICU location and sex (Figure 3). The patients in the highest quartile (≥ 40 kcal/kg/day) therefore had an approximately four-fold increase in risk for BSI, compared with patients in the lowest quartile (≤ 25 kcal/kg/day).

We explored possible explanations for some patients receiving seemingly excessive amounts of parenteral calories. First, we sought to determine whether a higher parenteral caloric intake might be associated with a lower volume of or an absence of enteral nutritional support. We found that the average daily enteral calories and the number of patients receiving any enteral calories were similar across the maximum parenteral caloric quartiles, suggesting little, if any, effect of enteral support on BSI risk. We did observe, however, a substantial day-to-day variation in parenteral caloric intake that was greater in the patients who did develop BSI. For example, the range of daily parenteral calories (maximum minus minimum daily total) was 29 ± 12 kcal/kg in patients with BSI versus 25 ± 11 kcal/kg in patients without BSI. Finally, when comparing average rather than maximum parenteral caloric intake, we still observed a higher risk for BSI in association with increased average parenteral calories. Taken together, this information suggests that increased parenteral calories, however quantified, are related to increased BSI risk.

We then focused on the patients in the highest quartile of parenteral caloric intake (≥ 40 kcal/kg) in order to determine whether and how they differed from the rest of the cohort. These patients were similar to the rest of the study population in terms of age, sex, admission diagnosis or reason for initiation of TPN. Factors contributing to higher maximum parenteral caloric intake were often unrelated to nutritional support *per se*. For example, in over one-half of the patients receiving ≥ 50 kcal/kg on at least 1 day, a considerable number of calories were given as 5% dextrose or propofol. While attempts were made to compensate for extraneous sources of calories when formulating TPN, this was not always successful. The highly variable rates of propofol infusion, for instance, caused patients to be overfed despite attempts at reducing the prescribed lipid calories. Dextrose calories were accounted for only when they were given at high rates of infusion, which may have led to underestimation of the impact of medications on the patient's calorie intake. Infusions of heparin and antihypertensive medications accounted for an unexpected calorie load in several patients. We do not routinely exceed the usual prescribed amount of TPN in order to 'catch up' where the regular TPN volume for the previous day had not been given. In a small number of cases, however, this appears to have occurred, resulting in unusually large caloric intakes the following day. In a minority of cases, adjustments were not made when patient weight estimates were corrected (downward) or when a relatively high stress factor multiplier (1.5 × basal energy expenditure) was used.

**Figure 4:**
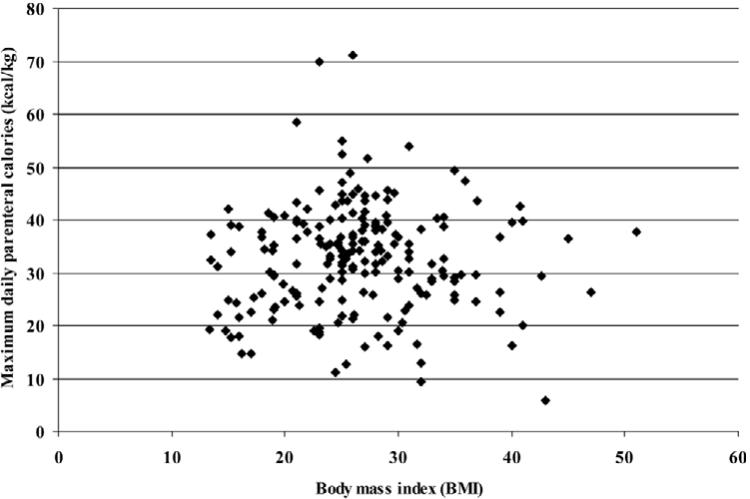
Maximum daily parenteral caloric intake versus body mass index. Maximum daily parenteral calories (kcal/kg adjusted body weight) plotted on the *y *axis against body mass index on the *x *axis. It is evident that patients with higher body mass index were no more likely to receive increased parenteral calories.

A substantial number of our patients were overweight or obese (51 patients (26%) had BMI ≥ 30 kg/m^2^). Given that estimated caloric needs were calculated differently for patients with BMI ≥ 30 kg/m^2^, we sought to determine whether this might influence our observed association between parenteral caloric intake and BSI. First, we observed the BSI risk to be slightly lower in patients with a BMI < 25 kg/m^2 ^(24/74 patients, 32%) than in patients with a higher BMI (52/132 patients, 40%). This difference was not statistically significant, however, and including the BMI in our logistic regression analysis of risk factors for BSI did not influence the relationship between parenteral caloric intake and BSI. Finally, as shown in Figure 4, there appears to be no relationship between parenteral caloric intake and BMI, suggesting that we did not simply give overweight and obese patients relatively more calories.

Contrasting our observations relating parenteral calories to BSI, we did not observe BG concentrations to be associated with infection risk. The median overall BG concentration was 134 mg/dl. The maximum BG values were similar in patients with BSI and those without BSI (197 mg/dl versus 196 mg/dl, respectively). We compared the BG level in each of the three time periods – morning, afternoon and evening – to examine whether the addition of lipid infusions at night had an impact on glycemic control. There was no difference in BG values between these time periods. We conducted analysis of variance to examine the effect of age, sex, hospital location (ICU versus ward), TPN duration and the presence of a pre-existing diagnosis of diabetes on BG values. BSI remained independent of hyperglycemia in this multivariate model.

Factors that appeared significant for BSI risk on univariate analysis – low plasma albumin, duration of nutrition and hospital length of stay – were not shown to be significant once adjustments for ICU location, age and sex were made. ICU location remained an independent predictor of increased infection risk (Table 4).

**Table 4:** Multivariate analysis of factors associated with bloodstream infection risk

Variable	Adjusted odds ratio	*P *value	95% confidence interval
Male sex	1.6	0.13	0.8–3.1
Intensive care unit location	3.2	0.001	1.7–6.2
Maximum parenteral calories	1.6	0.002	1.2–2.1

Data from forward stepwise logistic regression. In this analysis, maximum parenteral nutrition calories per kilogram (adjusted weight for patients with body mass index ≥ 30 kg/m^2^) are categorized into four groups (quartiles): ≤ 25 kcal/kg, 26–34 kcal/kg, 24–39 kcal/kg, ≥ 40 kcal/kg.

## Discussion

Our observations suggest that the incidence of BSI is related to the amount of parenteral calories that patients received, rather than to their BG concentrations. Patients with BSI in our study received a longer duration of and higher doses of parenteral nutrition. Despite having comparable glycemic control, patients with BSI received a significantly larger number of parenteral calories – when considering maximum calories, patients with BSI received approximately 5 kcal/kg/day more than patients without BSI.

Several other studies have suggested a link between overfeeding and adverse outcomes. The Veterans Affairs Total Parenteral Nutrition Cooperative Study Group that noted an increase in TPN-associated infections, for example, utilized an average caloric intake of 40 kcal/kg/day [6] – a rate significantly higher than current standard practice. Studies in trauma patients and critically ill patients have shown an increase in infections in TPN patients with higher rates of caloric intake [5,24]. Krishnan and colleagues, in a cohort of medical ICU patients, found that patients receiving between 33% and 65% of the recommended daily caloric intake had a higher survival to hospital discharge and had a reduced long-term ventilation compared with patients outside this range [25]. The SUPPORT study showed that increased calories were associated with increased mortality in patients with sepsis and acute respiratory failure [26]. These findings suggest that commonly used rates of energy delivery may be higher than optimal, especially among critically ill patients. Sandstrom and colleagues performed a randomized trial of TPN providing 120% of calculated metabolic needs versus intravenous glucose infusion in postoperative general surgical patients. They found that 20% of unselected patients in the TPN group were unable to tolerate the metabolic load, and that the patients suffered an increased rate of cardiopulmonary complications, prolonged mechanical ventilation and a 36% mortality rate [27].

The mechanism of this increase in complications is uncertain. Jeejeebhoy and McCowen and colleagues have asserted that the infection risk in TPN is directly related to hyperglycemia from overfeeding [28,29]. The association between parenteral nutrition and hyperglycemia in the causal pathway of infection has been widely assumed. Our data suggest this is not the case.

We observed no association between hyperglycemia and BSI. One possible explanation for this observation is that even the relatively good BG control we achieved is sufficient to mitigate any adverse effect due to greater degrees of hyperglycemia. It is possible that higher glucose concentrations would have contributed to an even greater risk for BSI. In keeping with this notion, Cheung and colleagues noted a four-fold increase in infection risk with mean BG over 165 mg/dl in patients receiving TPN [30]. Patients below this BG level did not show a significant increase in risk. In our study, the glycemic protocol resulted in a median BG of 134 mg/dl, while the median of the patient's maximum BG was 196 mg/dl. Although still higher than our target of 80–110 mg/dl, these levels are below historical targets of 200 mg/dl. The 1997 consensus statement from the American College of Chest Physicians, for example, recommends glucose control below 225 mg/dl [31]. Many of the reports of hyperglycemia in patients receiving TPN predate the era of strict BG control. Our glycemic control protocol may have been able to prevent a noticeable difference in BSI rates in our patients. Despite rather effective glucose control, 39% of patients receiving TPN developed at least one BSI, which is comparable with published reports.

We only measured the maximum BG value occurring during an 8-hour period, and cannot comment on the occurrence of hypoglycemia. Given that the objective of the present study was to examine potential associations with TPN rather than to audit our insulin use, we chose not to record lowest insulin concentrations. Similarly, it is unlikely that any measure of variability in glucose concentrations would uncover an adverse association with BSI that we did not detect with either the maximum or average BG concentrations we chose as potential risk factors. While we did not record the dose of insulin that was given to each patient, our study was conducted during a time period of uniform glucose control protocols in the ICU and acute care wards, with predetermined insulin doses prescribed for each BG level. In this observational study, the insulin dose will be so highly correlated with glucose concentrations that an independent analysis would be difficult. Furthermore, the general consensus in the published literature seems to be that glucose control, or a lack thereof, is more probably the factor associated with or contributing to adverse outcomes rather than some other effect of insulin.

Patients who received more than 40 kcal/kg/day were not intentionally overfed. Slight errors in body weight estimates and the use of higher than typical stress factor adjustments were uncommon, but contributed to some instances of increased parenteral intake. Failure to account for additional caloric sources when prescribing TPN, however, was a major contributor to increased parenteral calories. One source of excess calories was the infusion of propofol. There is evidence that excess intravenous lipids adversely affect immune function, and may be associated with increased infections and worse outcomes [32]. Although attempts were made to compensate for the excess lipids, the highly variable rate of propofol infusion resulted in an overestimation occurring most of the time. It would appear that intentionally leaving a larger 'window of error' and using lower caloric targets will help prevent this problem in future. Failure to account for calories in medications was another significant source of error, with most of these calories being delivered via continuous dextrose infusions. It should again be noted that our analysis herein focused on the maximum daily caloric intake, rather than the average amount of calories given during the entire period, and therefore reflects transient overfeeding but not persistent overfeeding. Even such transient increases in parenteral caloric intake are associated with BSI.

Regardless of the nutrient composition, it seems we must be attentive to all sources of intravenous calories. A recent study by Hise and colleagues found that patients in their surgical ICU received approximately 250 kcal/day via intravenous sources other than parenteral nutrition, highlighting the importance of recording all caloric intakes and adjusting the TPN intake accordingly [33]. Adjusting calories to the lower end of the calculated range may help prevent inadvertent overfeeding in clinical practice. This is especially true in critically ill patients, who receive a wide range of medications and who often have large fluctuations in measured weight during their hospital admission.

Permissive underfeeding has been proposed as a method to avoid the complications of overfeeding [34]. Most attention has focused on obese patients in the ICU [35]. In the only prospective randomized study in nonobese patients, McCowen and colleagues evaluated hypocaloric TPN as a means of reducing hyperglycemia and infectious complications in 48 patients [36]. The authors provided 1,000 kcal and 70 g protein per day, and compared their outcomes with patients given standard TPN formulations. They found no significant difference in the rate of hyperglycemia, infectious complications or mortality [36]. Their study, however, was underpowered to note a statistical difference in infections. There was no increase in adverse events in the hypocaloric group and, despite a reduced nitrogen balance compared with the patients on conventional TPN, these patients appeared to have similar clinical outcomes. A larger randomized trial of hypocaloric nutrition is needed to determine whether reducing caloric goals will reduce the incidence of infections in patients receiving TPN.

## Conclusion

Increased parenteral caloric intake is an independent risk factor for BSI in patients receiving TPN. This association appears unrelated to hyperglycemia. Based upon our observations, we suggest that parenteral caloric intake be prescribed judiciously and monitored closely, accounting for all intravenous caloric sources.

## Key messages

• Increased parenteral calorie load is associated with an increased risk of BSI.

• Careful attention to less obvious sources of caloric intake is essential to avoid overfeeding in patients receiving TPN.

## Abbreviations

BG = blood glucose; BMI = body mass index; BSI = bloodstream infections; ICU = intensive care unit; TPN = total parenteral nutrition.

## Competing interests

The authors declare that they have no competing interests.

## Authors' contributions

SD was responsible for the literature review, for data analysis and for drafting the manuscript. MS collected the data and critically revised the manuscript. GEOK was responsible for the concept and design, for statistical analysis and for critical revision of the manuscript. KW provided data management.
